# Quantifying the Effects of Photoperiod, Temperature and Daily Irradiance on Flowering Time of Soybean Isolines

**DOI:** 10.3390/plants3040476

**Published:** 2014-11-07

**Authors:** Elroy R. Cober, Daniel F. Curtis, Douglas W. Stewart, Malcolm J. Morrison

**Affiliations:** Agriculture and Agri-Food Canada, Eastern Cereal and Oilseed Research Centre (ECORC), 960 Carling Ave, Bldg 110, Ottawa, Ontario K1A 0C6, Canada; E-Mails: curtisseeds@bioflora.ca (D.F.C.); stewartdmej@gmail.com (D.W.S.); malcolm.morrison@agr.gc.ca (M.J.M.)

**Keywords:** *Glycine max*, soybean, flowering time, photoperiod, temperature, irradiance, phenology, modeling

## Abstract

Soybean isolines with different combinations of photoperiod sensitivity alleles were planted in a greenhouse at different times during the year resulting in natural variation in daily incident irradiance and duration. The time from planting to first flower were observed. Mathematical models, using additive and multiplicative modes, were developed to quantify the effect of photoperiod, temperature, photoperiod-temperature interactions, rate of photoperiod change, and daily solar irradiance on flowering time. Observed flowering times correlated with predicted times (R^2^ = 0.92, Standard Error of the Estimate (SSE) = 2.84 d, multiplicative mode; R^2^ = 0.91, SSE = 2.88 d, additive mode). The addition of a rate of photoperiod change function and an irradiance function to the temperature and photoperiod functions improved the accuracy of flowering time prediction. The addition of a modified photoperiod function, which allowed for photoperiod sensitivity at shorter photoperiods, improved prediction of flowering time. Both increasing and decreasing rate of photoperiod change, as well as low levels of daily irradiance delayed flowering in soybean. The complete model, which included terms for the rate of photoperiod change, photoperiod, temperature and irradiance, predicted time to first flower in soybean across a range of environmental conditions with an SEE of 3.6 days when tested with independent data.

## 1. Introduction

Eight genes, which influence time from planting to first flower, have been identified in soybean [*Glycine max* (L.) Merr.] to date: E1 and E2 [1], E3 [2], E4 [3,4], E5 [5], E6 [6], E7 [7] and E8 [8]. Two of these, E3 [9] and E4 [10] have been identified as phytochrome A genes while E2 has been identified as a GIGANTEA homolog [11] and E1 as an inhibitor of FT [12]. Alleles at these loci, in conjunction with photoperiod [13] and temperature [14], regulate the timing of flowering and maturity of soybean lines. Soybean is a facultative short day plant, so under non-inductive long days (>14 h) and high temperatures (25 to 30 °C) dominant E alleles delay flowering, with the exception of the E6 locus [14]. Lower temperatures reduced the delaying effect of E alleles under non-inductive photoperiods [14]. Isolines with dominant E alleles at two or three loci flowered earlier under low temperature (18 C) and non-inductive photoperiods compared to higher temperature (28 °C) with non-inductive photoperiods, but the time to flowering in both instances was still greater than the time to flower under inductive short photoperiods [14]. This result underscored the importance of the E genes in the adaptation of soybean lines to specific climates and highlighted the requirement for a thorough understanding of their function in controlling flowering.

The majority of work quantifying the effects of E genes on flowering has been done in growth cabinets under constant daylength and temperature or in the field during summer months when much of the phenological development to flowering is close to the summer solstice with relatively long and constant daylengths. If a soybean line is grown at a different latitude or time of the year, different photoperiods and rates of photoperiod change will be encountered. This is a consideration for countries like India, China, and Australia where soybeans can be grown during the winter months, which have short but increasing photoperiods [15,16,17]. For this reason, it is important to study the flowering time allele responses under increasing and decreasing day lengths.

Two approaches have been used to model temperature and photoperiod effects on soybean phenology. One approach is to sum over separate temperature and photoperiod functions [18,19,20,21,22] with a convenient coefficient for quantifying genetic differences for photoperiod sensitivity. Unfortunately, simple addition doesn’t account for temperature effects on photoperiod sensitivity as shown by Cober *et al*. [14] who added a function to account for temperature-photoperiod interactions. A temperature-photoperiod interactive term was also used by Yan and Wallace [23]. The other approach is to multiply temperature and photoperiod functions which would introduce temperature-photoperiod interactions automatically [24,25,26,27,28]. Accounting for the rate of photoperiod change [19] and low solar irradiance values remains un-quantified. It is not known if daily solar irradiance can be low enough to reduce photosynthate production to a level where phenological development is inhibited. In this study, an experiment was devised to expose soybean plants to large changes in day lengths and varying amounts of daily irradiance while measuring the time from planting to first flower. The objective of this study was to identify and quantify the effects of: photoperiod, rate of photoperiod change, and daily irradiance on the time from planting to first flower in soybeans isolines varying in alleles governing photoperiod sensitivity. Modeling time to first flower started with an existing model incorporating temperature and photoperiod and the new model described in this manuscript added a non-linear photoperiod response function, a rate of photoperiod change function, and a daily irradiance function.

## 2. Results and Discussion

**Figure 1:**
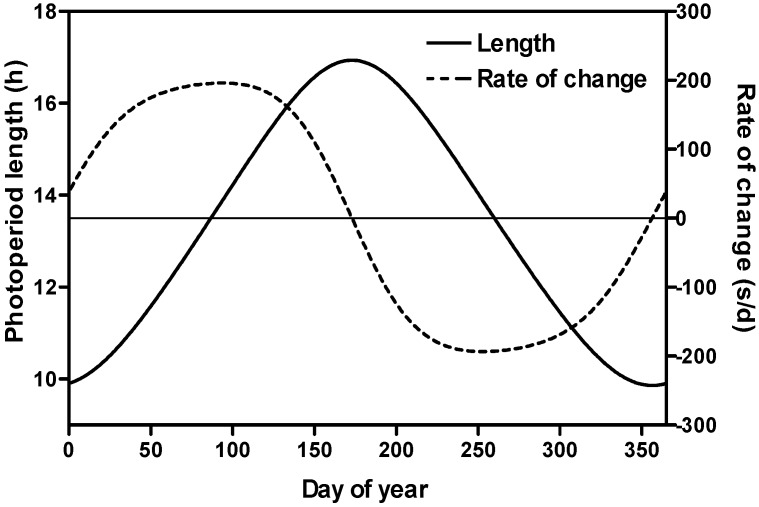
The rate of photoperiod change and the length of the photoperiod as it varied over a year at Ottawa, Canada.

**Figure 2:**
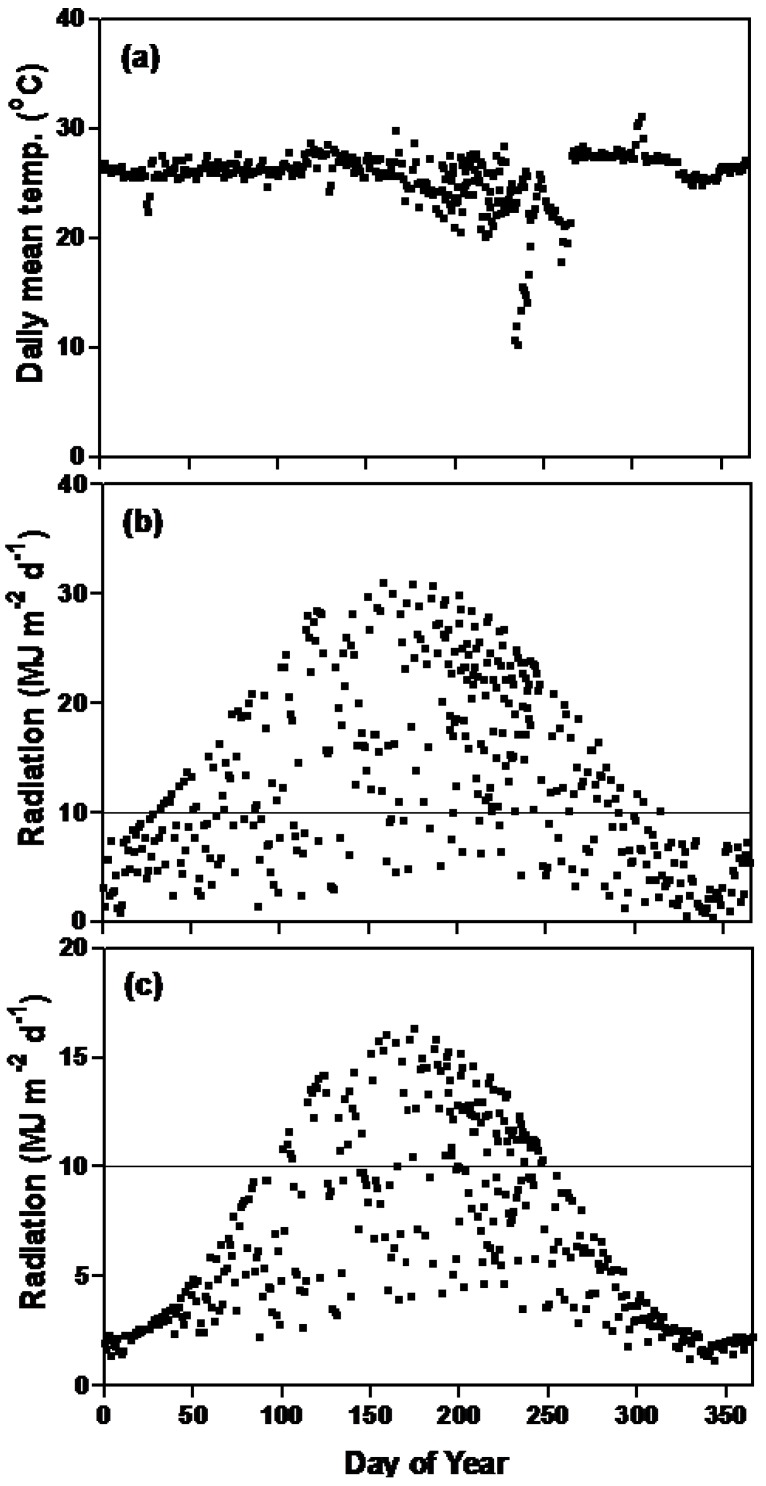
The (**a**) mean daily temperature in the greenhouse, (**b**) daily amounts of solar irradiance as measured at a weather station and (**c**) daily amounts of solar irradiance as calculated in the greenhouse during the study at Ottawa, Canada. The horizontal rule indicates 10 MJ m^−2^ d^−1^.

Our original soybean flowering time model had functions for temperature, photoperiod response and genotype specific parameters [29]. The soybean genotypes, maturity gene near-isogenic lines, used in this experiment shared one of two common genetic backgrounds “Harosoy” or “Clark” where combinations of early or later flowering alleles at various flowering loci were backcrossed into the recurrent parent (Table 1). Since we grew plants at a northern latitude with planting dates throughout the year, we produced a flowering time data set which resulted from growth of soybean isolines in a wide range of photoperiods as well as a wide range of rates of photoperiod change (Figure 1) and a range of incident irradiance (Figure 2b,c). Air temperature within the greenhouse was relatively constant except for some days in the late summer and early fall when supplemental heat at night was unavailable (Figure 2a).

**Table 1:** Harosoy (H) and Clark (C) soybean isolines used in the phenology study with their known maturity genotype and derived photoperiod coefficients with standard errors for the additive (c, Equation (14)) and multiplicative models (c*, Equation (15)). Dependent (Dep.) and independent (Ind.) photoperiod coefficients were derived from two data sets derived from alternate planting dates throughout the year.

Isoline		Maturity genotype ^†^	Photoperiod coefficient (standard error)			
			Additive (°C^−1^ d^−1^ h^−1^)		Multiplicative (h^−1^)	
			Dep.	Ind.	Dep.	Ind.
OT 94-47	H	e1e2e3e4e5e7	1.32 (0.12)	1.53 (0.18)	0.0206 (0.0057)	0.0193 (0.0098)
OT 89-5	H	e1e2e3e4e5E7	1.66 (0.13)	1.94 (0.23)	0.0416 (0.0068)	0.0478 (0.0116)
OT 93-26 ^‡^	H	E1e2e3e4e5E7	2.32 (0.09)	2.52 (0.15)	0.0806 (0.0048)	0.0822 (0.0069)
OT 93-28 ^‡^	H	E1e2e3e4e5E7	2.38 (0.09)	2.57 (0.15)	0.0839 (0.0045)	0.0859 (0.0070)
OT 94-41	H	e1e2E3e4e5E7	2.10 (0.09)	2.28 (0.14)	0.0675 (0.0050)	0.0686 (0.0079)
L92-21	C	e1e2E3e4e5E7	1.90 (0.12)	2.17 (0.18)	0.0543 (0.0063)	0.0609 (0.0092)
L62-667	H	e1e2e3E4e5E7	2.00 (0.12)	2.25 (0.18)	0.0624 (0.0067)	0.0663 (0.0104)
L71-920	C	e1e2e3E4e5E7	2.06 (0.10)	2.23 (0.16)	0.0657 (0.0054)	0.0650 (0.0084)
L71-802	H	E1e2e3E4e5E7	3.17 (0.05)	3.28 (0.07)	0.1304 (0.0029)	0.1290 (0.0046)
L80-5914	C	E1e2e3E4e5E7	3.13 (0.06)	3.18 (0.07)	0.1283 (0.0036)	0.1234 (0.0040)
L84-307	H	e1E2e3E4e5E7	2.71 (0.10)	2.81 (0.11)	0.1034 (0.0052)	0.1011 (0.0064)
L63-2404	C	e1E2e3E4e5E7	2.65 (0.07)	2.76 (0.09)	0.0996 (0.0038)	0.0972 (0.0052)
Harosoy	H	e1e2E3E4e5E7	2.25 (0.12)	2.44 (0.18)	0.0772 (0.0068)	0.0780 (0.0090)
L84-307	H	e1e2e3E4E5E7	2.10 (0.12)	2.28 (0.19)	0.0671 (0.0067)	0.0686 (0.0103)
L74-441	H	E1E2e3E4e5E7	3.63 (0.05)	3.73 (0.07)	0.1567 (0.0027)	0.1561 (0.0043)
L67-2324	H	E1e2E3E4e5E7	3.79 (0.06)	3.82 (0.04)	0.1662 (0.0029)	0.1615 (0.0031)
L66-432	C	E1e2E3E4e5E7	3.66 (0.09)	3.75 (0.09)	0.1588 (0.0050)	0.1569 (0.0054)
L64-4584	H	e1E2E3E4e5E7	3.17 (0.06)	3.19 (0.08)	0.1311 (0.0034)	0.1243 (0.0043)
Clark	C	e1E2E3E4e5E7	3.14 (0.07)	3.13 (0.09)	0.1294 (0.0041)	0.1203 (0.0054)
L64-4830	H	e1e2E3E4E5E7	3.22 (0.10)	3.12 (0.12)	0.1345 (0.0051)	0.1194 (0.0068)
L94-1110	C	e1e2E3E4E5E7	2.72 (0.15)	2.70 (0.16)	0.1042 (0.0082)	0.0930 (0.0092)
L71L-3004	H	E1E2E3E4e5E7	4.44 (0.05)	4.47 (0.07)	0.2034 (0.0030)	0.2001 (0.0042)
L65-3366	C	E1E2E3E4e5E7	4.23 (0.05)	4.31 (0.09)	0.1916 (0.0031)	0.1910 (0.0050)
L71L-3015	H	E1e2E3E4E5E7	4.21 (0.05)	4.28 (0.09)	0.1907 (0.0026)	0.1889 (0.0050)
L74-66	H	e1E2E3E4E5E7	3.12 (0.12)	3.36 (0.16)	0.1276 (0.0069)	0.1361 (0.0087)
L92-1195	C	e1E2E3E4E5E7	3.21 (0.12)	2.99 (0.13)	0.1310 (0.0069)	0.1114 (0.0080)

^†^ Dominant E alleles are late flowering under non-inductive long photoperiods while recessive e alleles are early flowering under non-inductive long photoperiods; ^‡^ These isolines have the same maturity genotype but differ for pubescence color.

### 2.1. Irradiance

**Figure 3:**
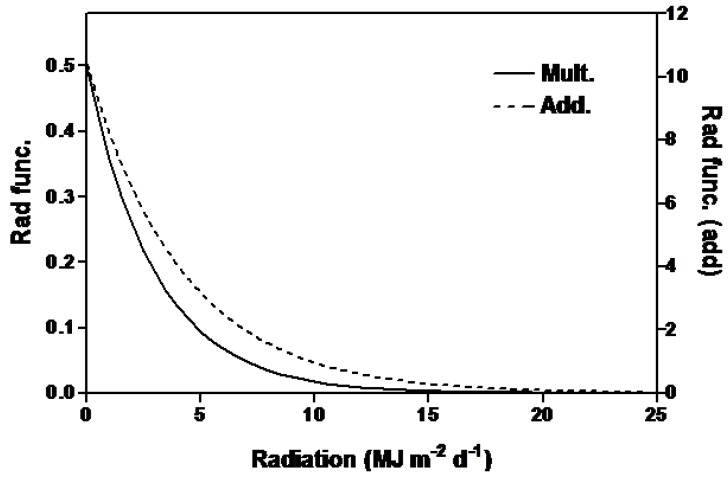
The solar irradiance functions for the multiplicative and additive models which predict flowering time in soybean.

**Figure 4:**
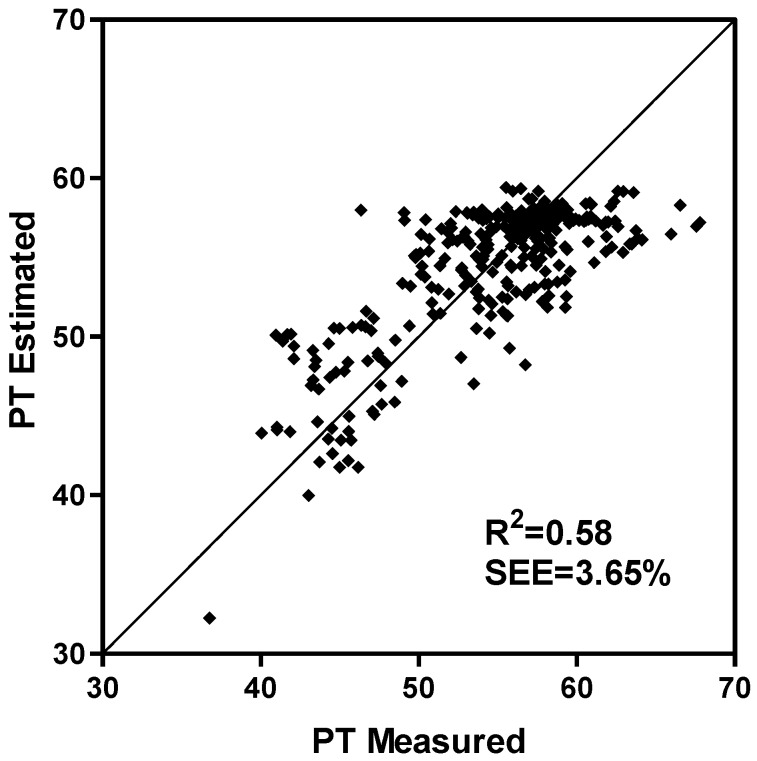
A comparison of measured and calculated percent transmission (PT; Equation (1)) of irradiance in the greenhouse used in the study of soybean flowering time at Ottawa, Canada.

As a result of a northerly location and winter conditions, we had some days with very low amounts of solar radiation (Figure 2b,c). As solar irradiance increased, the size of the irradiance function decreased exponentially in both the multiplicative and additive models (Figure 3) and as a result the value of the function quickly became small. For the conditions encountered in this experiment, the irradiance functions were important to the accuracy of the models because they reduced the SEEs by 2.24 and 3.77 d for the multiplicative and additive models, respectively (Table 2). An estimate, using Equation (1) (see Section 3, Experimental), of the percent incident irradiance transmitted through the greenhouse was made using daily solar irradiance measured at a weather station approximately 500 m from the greenhouse. In estimating irradiation, Equation (1) accounted for 58 percent of the variation between measured and calculated percent transmission into the greenhouse (P_T_) (Figure 4) with a relatively small SEE of 3.65%. Using Equation (1) was far superior to using an average percent transmission (data not shown [30]). Calculated irradiance values inside the greenhouse were about 50% of those outside the greenhouse. Within the greenhouse, irradiance was reduced during the winter months to about 20% of maximum irradiance in the summer (Figure 2b,c).

We found the addition of an irradiance parameter improved the model’s prediction of first flower. When the daily irradiance was less than 10 MJ m^−2^, flowering was delayed in our model. Birch *et al*. [31] studied phyllochron length (the time between successive leaf emergence) in maize (*Zea mays* L.) under shading in the field. They found a phyllochron increase of 2 to 4 °C d^−1^ MJ^−1^ of PAR irradiance, as it decreased from 9.6 to 1.1 MJ PAR m^−2^ d^−1^. Similarly increasing irradiance, to 20 h of 650 μmol m^−2^ s^−1^ radiation from 10 h of 650 μmol m^−2^ s^−1^ plus 10 h of 5–10 μmol m^−2^ s^−1^ radiation, increased the rate of leaf appearance in maize [32]. In a study of quinoa (*Chenopodium quinoa* Willd.) [33], seven cultivars which were sensitive to rate of change of photoperiod were also sensitive to irradiance, where lower irradiance increased the phyllochron. Horticultural researchers have been active in researching irradiance effects on development as it affects commercial greenhouse-produced ornamentals. In a study with yarrow (*Achillea millefolium* L. “Summer Pastels”), Zhang *et al*. [34] used controlled environments and found that irradiance levels of 100 µmol m^−2^ s^−1^ delayed anthesis 20 d compared to irradiance levels of 300 µmol m^−2^ s^−1^. Mattson and Erwin [35] examined time to flower, as a function of leaf number at first flower, for 41 species of herbaceous ornamentals grown from seed. In this work, short 8 h treatments of ambient irradiance ranging from 222 to 458 μmol m^−2^ s^−1^ were enriched with 50, 100, or 150 μmol m^−2^ s^−1^ additional irradiance, while long photoperiods were provided with the same short day irradiance plus an additional 10 h of 50, 100, or 150 μmol m^−2^ s^−1^ additional irradiance. Of the 41 species, one did not flower, and 28 were insensitive to irradiance, that is increased irradiance under inductive conditions did not reduce the number of leaves at first flower. Ten species showed a facultative irradiance response, that is, there were reduced leaf numbers at first flower as irradiance increased, while two species had increased leaf numbers at flowering as irradiance increased.

In our study, low irradiance delayed soybean flowering. To the best of our knowledge, irradiance functions have not been incorporated into soybean phenological models before this study. Part of the importance of the irradiance function, in our work, was due to the experimental conditions within the greenhouse, where a relatively large number of days were encountered where daily irradiance totals in the greenhouse fell below 10 MJ m^−2^ d^−1^ (Figure 2c) compared to amounts falling on the greenhouse (Figure 2b). During the summer months (days 130 to 270) of this experiment, there were relatively few days with less than 10 MJ m^−2^ d^−1^ of incident irradiance measured at the weather station. Therefore, for most field environments the irradiance effect would be negligible. In maritime climates or years with rainy, cloudy conditions in continental climates, and for some growth room and greenhouse experiments, lower incident solar irradiance could be a factor in determining phenology in soybean and other crops.

### 2.2. Rate of Photoperiod Change

In this experiment, the addition of the rate of photoperiod change function to the model had a relatively large effect in reducing SEEs by 1.31 and 2.66 d for the multiplicative and additive models respectively (Table 2). The rate of photoperiod change (RPC) coefficients (g and g*) were similar in value and the absolute values of their positive and negative components were not significantly different (Table 3). They have opposite signs to compensate for the change in sign of the rate of photoperiod change. We found that both increasing and decreasing rates of change of photoperiod delayed flowering.

The addition of the rate of photoperiod change (RPC) parameter improved the precision of our soybean flowering time model. Constable and Rose [19], working with a range of soybean cultivars, found the addition of a daylength rate of change parameter also improved their flowering time model. More studies of RPC effects on crop plant development have been done in cereals than in soybean. Kirby *et al*. [36], using nine cultivars of barley (*Hordeum vulgare* L.) found a negative RPC lengthened the phyllocron interval while a positive RPC shortened the phyllocron interval. In contrast, Kernich *et al*. [37] using two barley cultivars found that phyllochron and time to awn initiation was not affected by RPC. In wheat (*Triticum aestivum* L.), Baker *et al*. [38] worked with one cultivar and found the phyllchron was more correlated with RPC than the mean photoperiod and that a negative RPC delayed the phyllocron while a positive RPC increase the rate of leaf emergence. In contrast, Slafer *et al*. [39] found that the phyllochron and final leaf number, as well as time from emergence to terminal spikelet emergence [40] was not affected by RPC. In maize, a short day cereal, Bonhomme *et al*. [41] studied six cultivars and found only the tropical cultivars had significant response to RPC where leaf number was reduced (early flowering) with a positive RPC. Clerget *et al*. [42] studied three tropical sorghum (*Sorghum bicolour* (L.) Moench) cultivars, also short day cereals, and found conflicting results in controlled environments with different RPC. In field studies using different planting dates, they found that the addition of a RPC factor to their photoperiod model improved the prediction of panicle initiation and a negative RPC speeded flowering. We found that both a positive and negative RPC delayed soybean flowering. This is similar to results found in a study of RPC effects on phyllochron of quinoa where seven of nine cultivars studied were sensitive to RPC and both negative and positive RPC lengthened the phyllochron compared to nearly constant photoperiods [33]. There are many conflicting results when considering effects of RPC on either the phyllochron or phenology. An understanding of the effect of RPC is important when modeling plant growth across a range of environmental conditions.

**Table 2:** Model standard errors of estimate (SEE) and R^2^ for complete and reduced models for predicting time to first flower for soybean.

Model		Multiplicative		Additive	
	N	R^2^	SEE (d)	R^2^	SEE (d)
Complete model ^†^ (full dataset)	389	0.92	2.84	0.91	2.88
Without non-linear photoperiod function	389	0.90	3.18	0.89	3.34
Without rate of photoperiod change function	389	0.90	4.15	0.87	5.54
Without irradiance function	389	0.87	5.08	0.84	6.64
Without all of the above three functions	389	0.80	7.46	0.75	10.10
Complete model (dependent data)	207	0.92	2.53	0.93	2.89
Complete model (independent data)	187	0.89	3.61	0.90	3.55

^†^ Model with temperature, non-linear photoperiod, rate of photoperiod change, and daily irradiance functions, and a photoperiod coefficient for each isoline.

**Table 3:** Non-photoperiod coefficients for modeling soybean flowering time (with standard errors (SE) and units) obtained by least squares fitting to observations of times from planting to first flower from a full (N = 389) and reduced (N = 207) dataset. Coefficients are found in Equations (7), (11), (13) and (14) (additive model) and Equations (9), (12) and (15) (multiplicative model). Multiplicative model coefficients are denoted with an * while additive model coefficients do not have an *.

N	Multiplicative Model				Additive Model			
	Coefficient	Value	SE	Units	Coefficient	Value	SE	Units
389	b *	0.0021	3.4 × 10^−5^	°C^−1^ d^−1^	b	0.0024	3.8 × 10^−5^	°C^−1^ d^−1^
389	g * (plus)	9.464	1.080	dh^−1^	g (plus)	11.40	0.983	dh^−1^
389	g * (neg)	−9.822	1.078	dh^−1^	g (neg)	−11.02	0.921	dh^−1^
389	h *	0.499	0.060	h^−1^	h	10.20	0.438	^O^C h^−1^
389	k *	0.333	0.066	d J^−1^	k	0.228	0.023	d J^−1^
207	b *	0.0021	4.2 × 10^−5^	°C^−1^ d^−1^	b	0.0025	3.7 × 10^−5^	°C^−1^ d^−1^
207	g * (plus)	7.975	1.461	dh^−1^	g (plus)	12.92	0.804	dh^−1^
207	g * (neg)	−11.62	1.353	dh^−1^	g (neg)	−14.64	0.795	dh^−1^
207	h *	0.460	0.050	h^−1^	h	11.94	0.484	°C h^−1^
207	k *	0.297	0.068	d J^−1^	k	0.302	0.018	d J^−1^

### 2.3. Photoperiod

**Figure 5:**
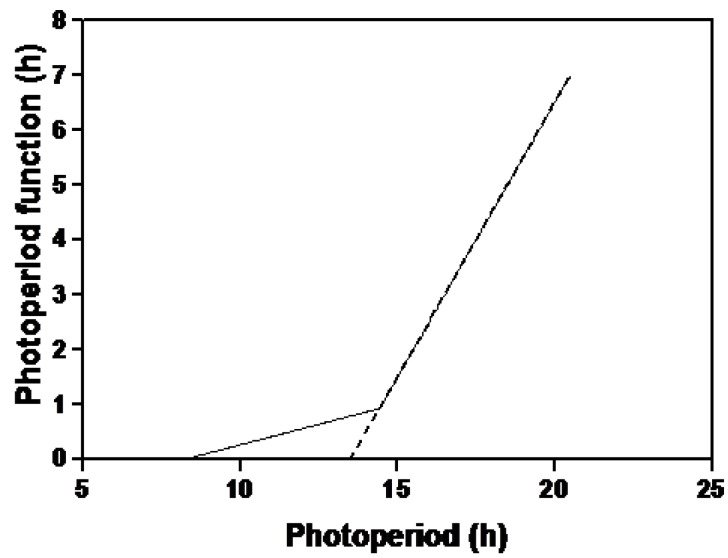
The original one-line photoperiod function (broken line) and the modified two-line function (solid line) which was used in this study for modeling soybean flowering time.

The small effect of the non-linear photoperiod response (Figure 5) was unexpected as it first appeared that we were obtaining large photoperiod responses (flowering delays) at photoperiods less than 13.5 h. These delays occurred during the short, cloudy days of late fall and the introduction of the irradiance function accounted for these flowering delays much better than the two-line photoperiod response. Our initial photoperiod function had the photoperiod effect decline to nil at 14.3 h.

Our current work has showed that a two line function which allowed a photoperiod effect at shorter photoperiods, as low as 8.2 h, somewhat improved our ability to predict flowering time. Sinclair *et al*. [43] working with long juvenile and “normal” lines found the normal line showed a response to photoperiod as it lengthened from 11.5 to 14.4 h. Grimm *et al*. [25] modeled optimum night length, at which rate of development to flowering was fastest, using cultivars ranging from maturity group 000 to VII. When night length was converted to photoperiod, optimum photoperiods were found to be 13.6 to 14 h for the earliest three maturity groups and 12 to 12.8 h for the latest three maturity groups. Grimm *et al*. [25] found that there were significant differences for optimum night length among cultivars. While most soybean crops will not be grown in photoperiods much shorter than 12 h, very short photoperiods could be experienced by subtropical, winter-grown crops.

### 2.4. Model Development and Extension

**Figure 6:**
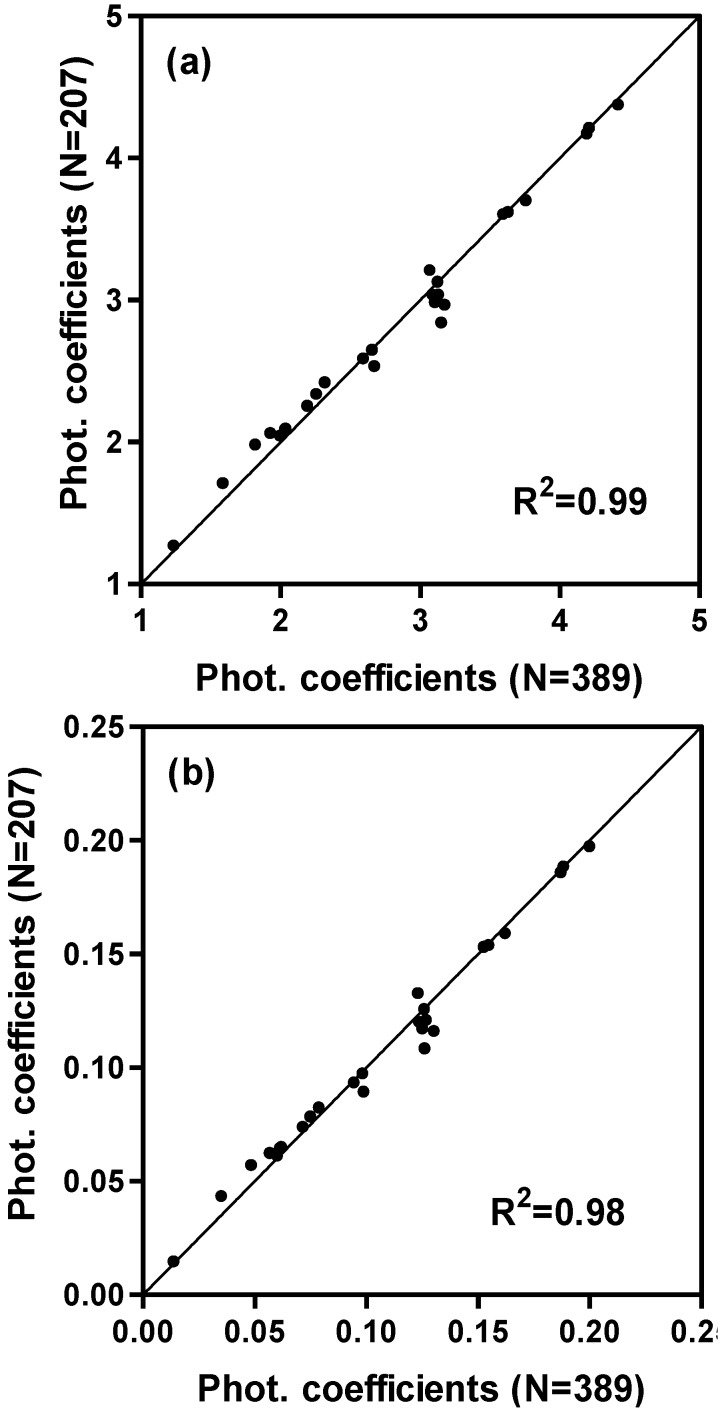
Comparisons of the photoperiod coefficients for the (**a**) additive model (c (°C^−1^ h^−1^)) and for the (**b**) multiplicative model (c* (h^−1^)). These coefficients were calculated by least squares using the whole data set (N = 389) and using the subset (N = 207).

When developing these models, we used the whole data set. To verify the independence of the model we divided the data set nearly in half with alternating planting dates assigned to serve as approximations of dependent and independent data sets. The dependent half of the data (n = 207) was used to re-solve for the coefficients in the model. The coefficients generated from the dependent half data set were approximately the same as in the initial set when all the data (n = 389) was used (Table 4 and Table 3, Figure 6). When these dependent half-data-set coefficients were used in the model and the model applied to the independent half of the dataset (n = 187), the SEE increased by about a day from the original dependent calculations (Table 2). While there are small differences in coefficients between dependent and independent solutions, the conclusions drawn from the complete dataset appear valid.

**Table 4:** Values and standard errors (SE) of the empirical “a” coefficients of Equation (1) which were used to calculate percent transmission of incident radiation into the greenhouse used in the study of soybean flowering time.

Coefficient	Value	SE	Units
a_1_	61.17	1.81	%
a_2_	−25.47	2.20	%
a_3_	−0.061	0.039	% °C ^−1^
a_4_	0.439	0.050	% °C ^−1^

**Figure 7:**
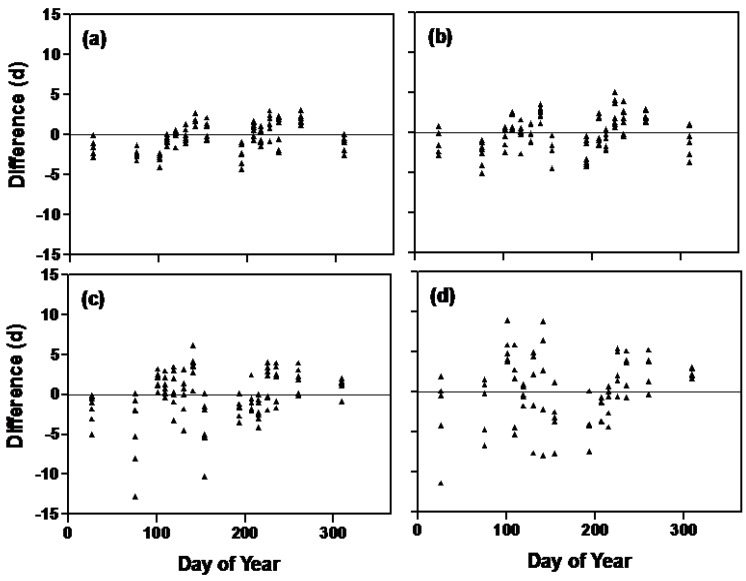
Differences between multiplicative model predictions and observations for soybean isoline flowering time as they varied with planting day of the year for all 26 isolines. Isolines increase in photosensitivity from (**a**) to (**d**). The first seven isolines from Table 1 are grouped in (**a**) (OT 94-47, OT 89-5, OT 93-26, OT 93-28, OT 94-41, l92-21, l62-667), the next seven in (**b**) (L71-802, L80-5914, L84-307, L63-2404, Harosoy, L84-307), the next seven in (**c**) and the last five in (**d**).

When plotting the difference between observed and predicted (using the multiplicative model) days to first flower, we found patterns with day of the year (Figure 7). The differences increased with the photosensitivity of the lines (*i.e*., from a to d). The same pattern occurred with the additive model (data not shown [30]). This indicated that not all the planting date to flowering time variation was accounted for in these models. This may not be surprising because we did not measure the actual irradiance each plant received. Thus, there could have been azimuthal effects caused by the geometry of the greenhouse and sun angles as they varied throughout the year. In any event the effects are not large, since SEEs of these models were less than three days.

**Figure 8:**
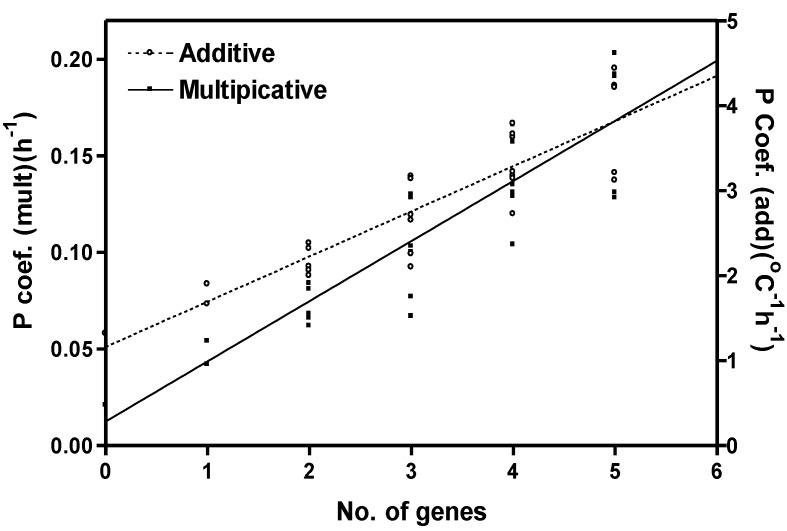
Photoperiod coefficients for soybean isolines (Y) for the multiplicative and additive models as related to the number of loci (X) in each isoline with dominant (late flowering) genes. For the multiplicative model, the equation is Y = 0.013 + 0.031X, R^2^ = 0.79, SEE = 0.023 h^−1^. For the additive model, the equation is Y = 1.15 + 0.531X, R^2^ = 0.79, SEE = 0.389 °C h^−1^.

Since the near-isogenic lines share two common backgrounds, with alternative early or late flowering alleles across a range of loci, we were also interested in flowering-time gene effects. To investigate on a gene, rather than isoline basis, multiplicative and additive photoperiod coefficients were plotted for each isoline based on the number of loci with late flowering alleles, *i.e*., the number of late flowering genes (Figure 8). Based on this classification, the isolines ranged from having zero late flowering alleles (early flowering genotypes) to having late flowering alleles at five loci (late flowering genotypes). The effect of late flowering genes appears additive although some non-additivity was seen especially with larger numbers of late flowering genes.

## 3. Experimental

### 3.1. Plant Experiments

“Harosoy” and “Clark” near-isogenic soybean lines with different combinations of alleles at six E loci influencing photoperiod sensitivity were grown under greenhouse conditions. There were a total of 17 maturity genotypes in the Harosoy background with nine of these maturity genotypes duplicated in the Clark background (Table 1). E6 isolines were not available. Seeds were planted in vermiculite and seedlings were transplanted to 13 cm diameter pots following unhooking of the hypocotyl. The soil mix consisted of a sterilized mix in a ratio of 3:2:1:0.5:0.5 of loam soil, peat, sand, vermiculite, and crushed brick, respectively. Air temperature in the greenhouse was recorded once each hour during the study. Hourly temperatures were averaged to obtain a mean daily temperature. The date of the first open flower was recorded for each plant.

The plants were grown in a greenhouse under natural daylengths (Figure 1) in Ottawa, Ontario, Canada (45°23'N, 75°63'W). High pressure sodium lamps provided supplemental light when irradiance fell below 100 W m^−2^ outside the greenhouse and were limited to 1 h after sunrise and turned off 1 h before twilight so as not to affect the photoperiod. The output from the high pressure sodium lamps at the plant canopy level was 32.28 W m^−2^. The instantaneous readings were multiplied by the number of seconds the lamps were on during the day and the values added to the total irradiance calculated for the greenhouse.

The experiment consisted of 15 planting dates with three replications at each planting date. Replicates were spatially separated on different greenhouse benches. The first planting series occurred in 1999 on 17 July, 26 July, 3 August, 13 August, 23 August, 17 September and 5 November. The second planting series occurred in 2000 on 26 January, 15 March, 10 April, 18 April, 28 April, 9 May, 20 May and 2 June. The planting dates were chosen to form a paired arrangement in which the seedlings emerged during similar photoperiods, with the difference that the 1999 dates represented shortening days and 2000 dates represented lengthening days. The longest day length including civil twilight (defined as the period prior to sunrise or following sunset when the centre of the sun is geometrically 6 degrees below the horizon) occurred on 21 June 1999 at 16 h 56 min. The shortest day length including civil twilight occurred on 21 December 1999 at 9 h 51 min. Days to the first opened flower were recorded for each plant.

Photosynthetically active radiation (PAR) was calculated using an estimate of the percent irradiance transmitted through the greenhouse and daily solar irradiance measured at a weather station approximately 500 m from the greenhouse. To estimate percent PAR transmission, an experiment was devised in April 2004 where irradiance was measured continuously on a quantum basis, within the greenhouse using five 1-m line quantum sensors (Model Li-191SA, LICOR, Lincoln, NE, USA) set at five locations in the greenhouse at plant height. Average hourly values were determined. Irradiance was also measured outside the greenhouse with a quantum sensor (Model Li-190SA, LICOR, Lincoln, NE, USA). Percent PAR transmission into the greenhouse was affected by cloudiness and solar elevation (S_E_). Solar elevation was calculated from astronomical equations [44]. Cloudiness was represented by the fraction (F_1_) where irradiance was divided by the quantity of a maximum 2500 µmol m^−2^ s^−1^, multiplied by the SIN of the solar elevation (S_E_). On a clear day in summer at solar noon this fraction would be close to one while on a cloudy day it could fall to as low as 0.1. Percent transmission (P_T_) of radiation into the greenhouse was related to F_1_ and S_E_ by:

*P_T_ = (a_1_ + a_2_F_1_) + (a_3_ + a_4_F_1_)S_E_* (1)

The empirical coefficients a_1_ to a_4_ were calculated by least squares from the values of P_T_, F_1_ and S_E_ generated from the greenhouse transmissivity experiment (Table 4).

Since irradiance was measured on an energy basis (W m^−2^) at a nearby weather station during the phenology experiment (1999 to 2000), and measured on a quanta (µmol m^−2^ s^−1^) basis during the transmissivity experiment, the cloudiness fraction during the phenological experiment (F_2_) was calculated for each hour of this experiment by dividing irradiance by the quantity of 1250 W m^−2^ times the SIN of S_E_. To relate the two irradiance measures, we assumed that approximately 1250 W m^−2^ of irradiance wouldbe measured as 2500 µmol m^−2^ s^−1^ using a quantum sensor. This also assumed that F_1_ (calculated on an energy basis during the percent transmissivity experiment) was equivalent to F_2_ (calculated throughout the phenology experiment) since visible and solar radiation have the same transmissivity through glass. Percent transmission into the greenhouse was calculated for each hour of the phenology experiment using Equation (1) with F_2_ substituted for F_1_. P_T_ multiplied by the hourly values of irradiance recorded at the weather station during the phenology experiment predicted hourly values of irradiance at the plant canopy level which were summed each day. Daily irradiance sums from the supplemental lamps were also added to daily irradiance values.

### 3.2. Theoretical Considerations for Modeling

In formulating a new model to predict time to first flower, we began with the mathematical model developed by Stewart *et al*. [29] which simulates time from planting to first flower from the average daily temperature, and latitude. The rate of change of phenological development was expressed as:

*dD/dt = b[(T − T_B_) − C_F_P_F_]* (2)

where D was phenological development, t was time in days, T was average daily air temperature, T_B_ was a base temperature below which temperature had no effect on development, b was an empirical coefficient, C_F_ was a temperature-photoperiod interaction function and P_F_ was a photoperiod function. The photoperiod function was defined following Stewart *et al*. [29] and Cober *et al*. [14] as:

*P_F_ = (P − 13.5) P_F_ = 0 when P < 13.5* (3)

P_F_ is illustrated in Figure 5. The function C_F_ was defined in Stewart *et al*. [29] and Cober *et al*. [14] as:

*C_F_ = c − d(T_D_ − T)* (4)

where c was a photoperiod coefficient for each isoline (Table 1), d was an interactive coefficient, and T_D_ was a daily mean temperature limit. C_F_ had the restriction that it must be greater than or equal to zero. In this model, the interactive term (d(T_D_-T)) increased as T fell below T_D_. When the temperature decreased to T_B_, photoperiod sensitivity disappeared where:

*T_B_ =T_D_ − d/c* (5)

From a previous study where values of T were changed systematically, T_D_ was set to 28 °C but could vary from 24 to 30 °C with compensating changes in d, an empirical coefficient [14].

Following Cober *et al*. [14], we assumed that D (phenological development) increased from zero to one from seeding to first flower. Using this assumption, Equation (2) can be expressed in integral form as:

(6) $$\int_{i=1}^{N}b[(T-T_{B})-C_{F}P_{F}]dt=1$$

where N was the number of days from planting to first flower. Dividing by b and integrating numerically resulted in a photothermal unit (G_PDD_) expressed as:

(7) $$G_{PDD}=\sum_{i=1}^{N}[(T-T_{B})-C_{F}P_{F})]\Delta t=1/b$$

where t was a time step of one day.

In a multiplicative approach, the rate of phenological development was expressed as:

dD/dt =b*[(T − T_B_)(1 − c*P_F_)] (8)

where b* and c* were the multiplicative equivalents to b and c. The multiplicative photo thermal equation was expressed as:

(9) $$G_{PDD}=\sum_{i=1}^{N}[(T-T_{B})(1-c*P_{F})]\Delta t=1/b*$$

In the current paper, the above models were modified in three ways. The first modification was to change the photoperiod function to P_FM_ so that photoperiod sensitivity would start at lower photoperiods. P_F_ was defined as

*P_FM_ = 0.13(P − 8.2) P_FM_ = 0 when P < 8.2* (10)

*P_FM_ = P_F_ when P > 14.3*

When the photoperiod was greater than 14.3 h, P_FM_ was set equal to P_F._ In other words, a one line function for photoperiod response was replaced with a two line function (Figure 5). Values for the coefficients in Equation (10) (0.13 and 8.2 h) were determined by least squares which is explained more fully below when we discuss the evaluation of all coefficients.

The second modification was to change the photoperiod functions to account for effects of the rate of photoperiod change. For the additive equation this was expressed as:

*C_FM1_ = c[1 + g(ΔP/Δt)] − d(T_D_ − T)* (11)

where ΔP/Δt was the rate of photoperiod change, and g was the rate of photoperiod change coefficient. The equivalent multiplicative equation was:

(12) $$C_{FM1}^{*}=c*[1+g*(\Delta P/\Delta t)]$$

The final modification was to add an irradiance function (R_F_) to the equations expressed as:

*R_F_ = h* exp*(−kR)* (13)

where R was the daily total irradiance. The hypothesis was that during some days, low amounts of irradiance lowered photosynthate production to a level where the rate of phenological development was reduced. The irradiance function has a maximum value h at zero daily irradiance but falls exponentially to near zero as irradiance increases. The C_F_ functions were changed to:

*C_FM2_ = c[1 − g(ΔP/Δt)] − d(T_D_ − T) − R_F_* (14)

and

(15) $$C_{FM2}^{*}=c*[1-g*(\Delta P/\Delta t)]-R_{F}^{*}$$

where $R_{F}^{*}$ was the corresponding equation for the irradiance function with h* and k*.

The final version of the additive model was expressed as:

(16) $$G_{PDD}=\sum_{i=1}^{N}[(T-T_{B})-C_{FM2}P_{FM})]\Delta t=1/b$$

The final version of the multiplicative model was expressed as

(17) $$G_{PDD}=\sum_{i=1}^{N}[(T-T_{B})(1-C_{FM2}^{*}P_{FM})]\Delta t=1/b*$$

To summarize, Equations (16) and (17) modeled flowering time using temperature, non-linear photoperiod, rate of photoperiod change and daily irradiance functions, and a photoperiod coefficient for each isoline. These two models are labeled complete models throughout this report compared to reduced models where functions are deleted from the complete models.

Following the methods of Stewart *et al*. [29], we assumed that c and c* varied with isoline but all other coefficients were constant with isoline. We assumed T_b_ and d were 5.78 and 0.196 respectively from Cober *et al*. [14] and Stewart *et al*. [29]. Values for b, b*, c, c*, f, f*, g, g*, h, h*, k and k* were calculated by least squares by fitting Equations (7) and (9) to observations of days to planting from the experimental data of this study using a non-linear least squares algorithm [45]. Two values each of g and g* were determined; one for increasing day length and one for decreasing day length. A value for c and c* was determined for each of the 26 isolines used in this study. Since Equation (10) was discontinuous and also had a relatively small effect on model calculations, we determined the values of 0.13 and 8.2 h by using simple iteration. That is, we ran the least squares algorithm for a range of values for the two coefficients. Values of 0.13 and 8.2 h resulted in the minimum sums of squares of model calculations and observation differences.

Models were evaluated using the coefficient of determination (R^2^) and standard error of estimate (SEE) defined by:

(18) $$SEE=\sqrt{\sum_{i=1}^{N}{\left(X_{C}-X_{O}\right)}^{2}/N_{DF}}$$

where N was the number of observations, X_C_ calculated values, X_O_ observed values and N_DF_ the number of degrees of freedom. We also plotted differences between model calculations and observations of days from planting to first flower as a function of day of year to determine if systematic differences were evident.

The effects of the modifications were determined by deleting the irradiance function, the rate of change function, and the non-linear photoperiod response function individually in turn and determining R^2^ and SEEs for each model where a function was deleted.

To address concerns for independent validation of the model, we tested the complete models further by dividing the data into two almost equal groups using alternating planting dates to sub divide the data set, for example, data from the 17 July and 3 August planting dates were in set 1 and data from 26 July and 13 August planting dates were in set 2. In one group we resolved for all the coefficients and then compared model calculations using the new set of coefficients with observations from the second independent data set. The parameters derived from the two data sets are shown in Table 1 as dependent (first dataset) and independent (second dataset). These datasets are truly independent since the environmental conditions experienced by the plants in each set are different.

## 4. Conclusions

**Figure 9:**
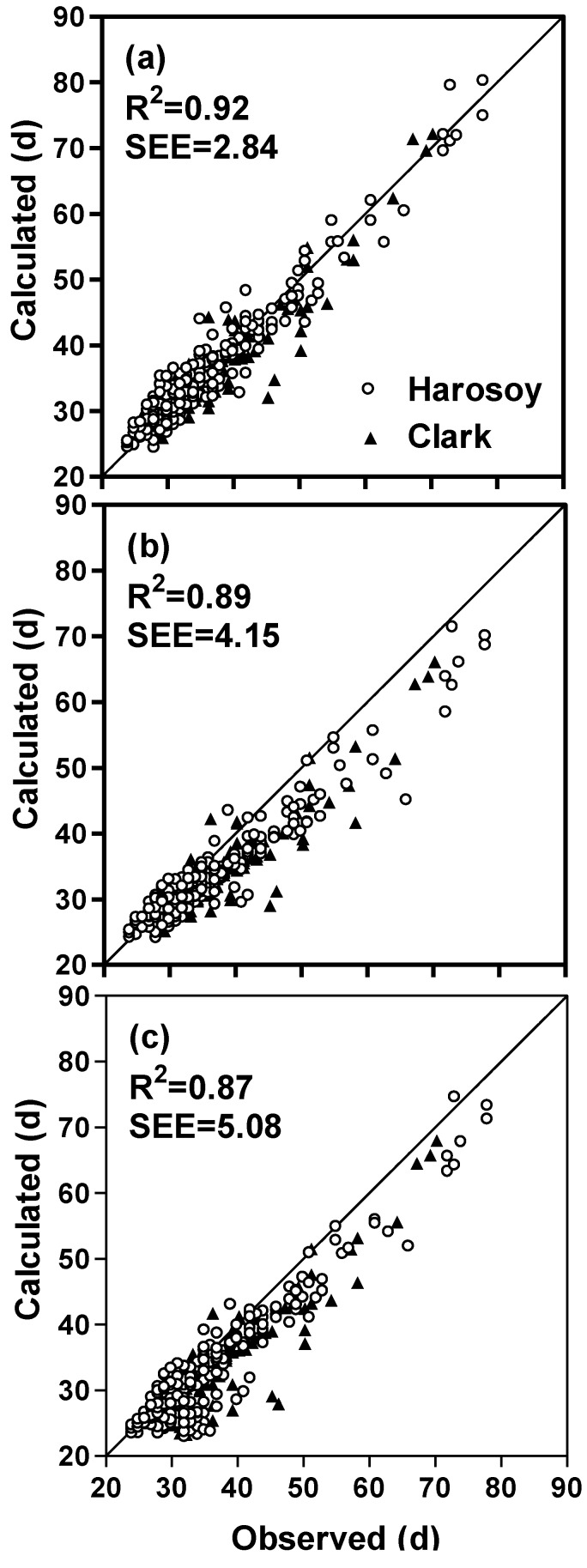
A comparison of calculated and observed days from planting to first flower for Harosoy and Clark soybean isolines for (**a**) the complete multiplicative model, (**b**) the complete model without the rate of photoperiod change function, (**c**) the complete model without the irradiance function.

**Figure 10:**
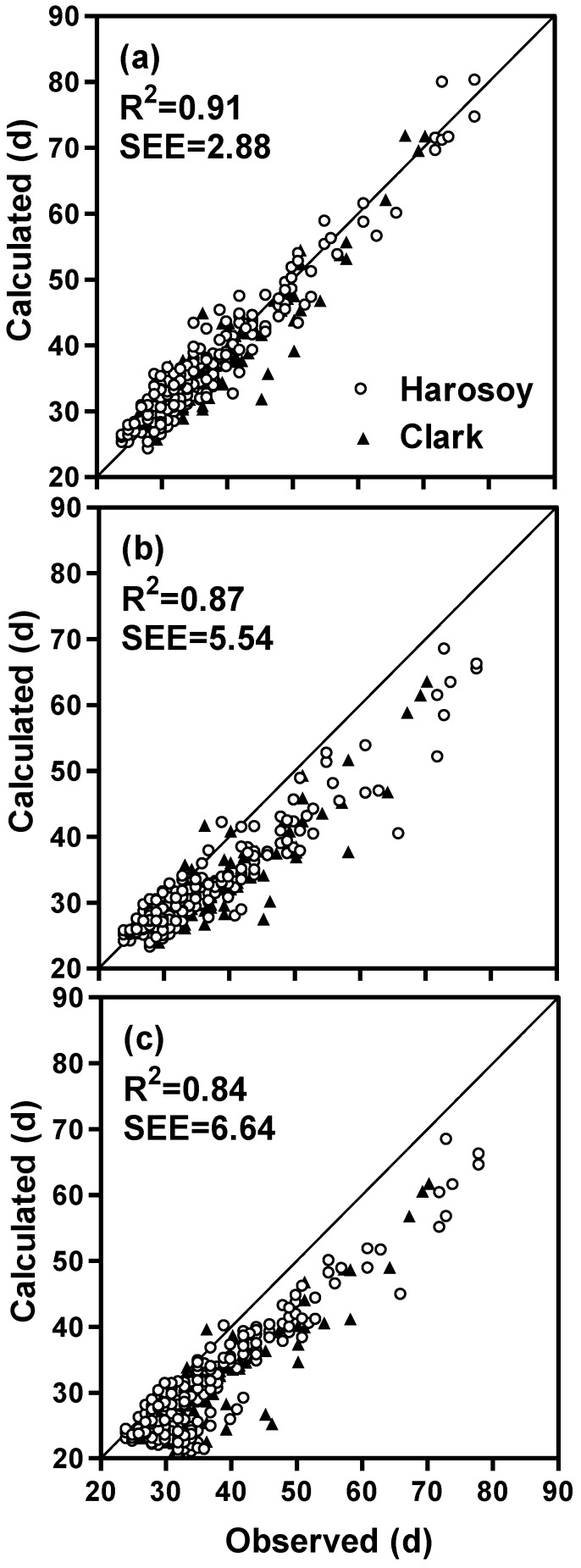
A comparison of calculated and observed days from planting to first flower for Harosoy and Clark soybean isolines for (**a**) the complete additive model, (**b**) the complete model without the rate of photoperiod change (**c**) the complete model without the irradiance function.

**Figure 11:**
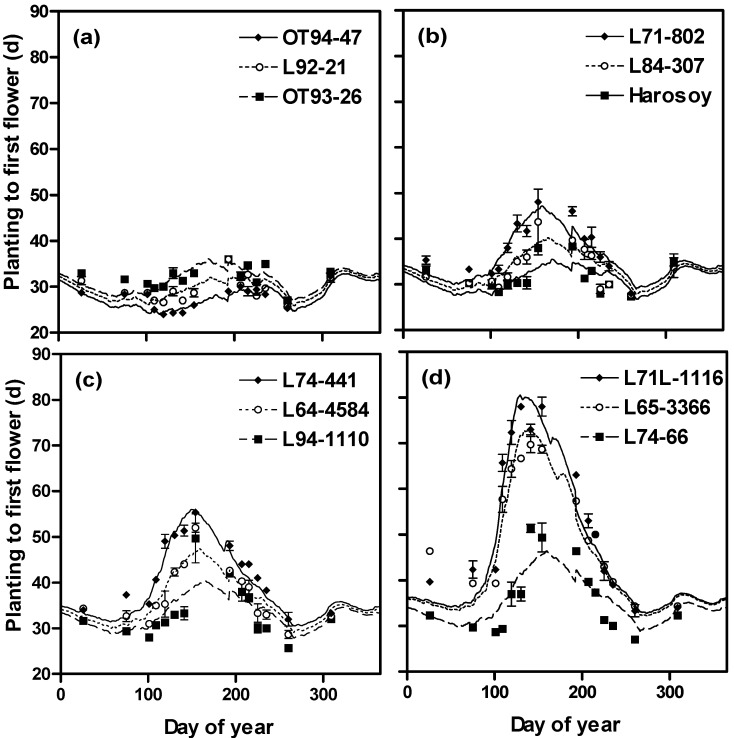
Days from planting to first flower *versus* the day of year when soybeans were planted based on the multiplicative model (lines) and observed values (symbols) for 12 soybean isolines varying from (**a**) least to (**d**) greatest photosensitivity.

The complete model (model with temperature, non-linear photoperiod, rate of photoperiod change and daily irradiance functions, and a photoperiod coefficient for each isoline) predicted days to first flower in agreement with observations, with R^2^ and SEEs of 0.92 and 2.84 d for the multiplicative model and 0.91 and 2.88 d for the additive model (Figure 9a, Figure 10a, Table 2). These SEEs compared well with the average standard deviation of the observed flowering times which was 0.97 days. The model was able to account for wide ranges in photoperiod responses throughout the year (Figure 11). Our original soybean flowering time model which did not contain an irradiance function, a rate of photoperiod change function, nor a non-linear photoperiod function had a much higher standard error of estimate for flowering time (mean of 8.8 d for both multiplicative and additive models, Figure 9b,c and Figure 10b,c) compared to our complete model with the addition of these three functions (mean of 2.9 d, Table 2). Rating the improvements generated by the individual functions showed that the largest improvement resulted from the addition of the irradiance function, followed by the rate of photoperiod change function, with the smallest improvement resulting from the addition of the non-linear photoperiod function (Table 2). The rate of photoperiod change can be a factor impacting soybean phenology, especially near the equinoxes, when photoperiod changes are the greatest. Surprisingly both increasing and decreasing photoperiods delayed flowering. Low (<10 MJ m^−2^ day^−1^) daily irradiance also delayed flowering. Lowered solar irradiance resulting from cloudiness could be a minor factor in Maritime climates or in cloudy years in continental climates.
